# METTL3 promotes osteoblast ribosome biogenesis and alleviates periodontitis

**DOI:** 10.1186/s13148-024-01628-8

**Published:** 2024-01-24

**Authors:** Yiwen Zhang, Yiping Kong, Wenjie Zhang, Jinlin He, Zhanqi Zhang, Yongjie Cai, Yiqing Zhao, Qiong Xu

**Affiliations:** 1 Hospital of Stomatology, Guanghua School of Stomatology, Guangdong Provincial Key Laboratory of Stomatology, Sun Yat-sen University, 56 Ling Yuan Xi Road, Guangzhou, 510055 China; 2 Peking University Shenzhen Hospital, Shenzhen, 518036 China; 3 Changsha Stomatological Hospital, Hunan University of Chinese Medicine, Changsha, 410004 China

**Keywords:** Periodontitis, Osteoblast, Ribosome biogenesis, METTL3

## Abstract

**Background:**

Periodontitis is a highly prevalent oral disease characterized by bacterium-induced periodontal inflammation and alveolar bone destruction. Osteoblast function is impaired in periodontitis with a global proteome change. METTL3 is the pivotal methyltransferase of *N*^6^-methyladenosine (m^6^A) that is recently proved to exert a crucial role in osteoblast differentiation. This study aims to investigate the role of METTL3 in osteoblast ribosome biogenesis in periodontitis progression.

**Results:**

METTL3 was knocked down in osteoblasts, and the downregulated genes were enriched in ribosome and translation. METTL3 knockdown inhibited ribosome biogenesis and oxidative phosphorylation in LPS-stimulated osteoblasts, whereas METTL3 overexpression facilitated ribosomal and mitochondrial function. Mechanistically, METTL3 mediated osteoblast biological behaviors by activating Wnt/β-catenin/c-Myc signaling. METTL3 depletion enhanced the mRNA expression and stability of Dkk3 and Sostdc1 via YTHDF2. In periodontitis mice, METTL3 inhibitor SAH promoted alveolar bone loss and local inflammatory status, which were partially rescued by Wnt/β-catenin pathway activator CHIR-99021 HCl.

**Conclusions:**

METTL3 promoted ribosome biogenesis and oxidative phosphorylation by activating Wnt/β-catenin/c-Myc signaling in LPS-treated osteoblasts and alleviated the inflammatory alveolar bone destruction in periodontitis mice.

**Supplementary Information:**

The online version contains supplementary material available at 10.1186/s13148-024-01628-8.

## Background

Periodontitis is an oral inflammatory disease that progressively impairs the supportive structures of the teeth. Initiated by bacterial biofilms, periodontitis is characterized by gingival inflammation and osteolytic lesions [1]. Lipopolysaccharide (LPS) derived from Gram-negative bacteria could disrupt the balance of bone resorption and formation and is considered as a vital inducer of inflammatory bone erosion in periodontitis [2, 3]. As the most important bone-forming cell, osteoblast plays a pivotal role in alveolar bone development, growth, and maintenance. The impairment of osteoblast function by periodontal inflammation is responsible for alveolar bone loss, which has been an ongoing challenge for periodontitis therapy [4, 5].

Most cellular dysfunctions, such as inflammation and tumorigenesis, are coordinated with ribosome-dependent proteome reshaping [6–8]. Ribosome forms the core of the translation machinery to decode mRNA messages and build proteins. The eukaryotic ribosomes are composed of different ribosomal RNAs (rRNAs) and ribosomal proteins (RPs), with the input of all three RNA polymerases (RNA pols) [9, 10]. Ribosome biogenesis is a multi-step process that begins in the nucleolus and concludes in the cytoplasm. The process is tightly controlled by multiple checkpoint and surveillance pathways [11]. Any disruptions of ribosome neosynthesis can lead to congenital disorders, including defects in the craniofacial, axial and limb skeleton [12]. Recent report showed that the ribosome biogenesis is dynamically orchestrated during osteoblast differentiation [13]. Nevertheless, the correlation between ribosome biogenesis and alveolar bone loss in periodontitis is still elusive.

*N*^6^-Methyladenosine (m^6^A) is one of the most abundant RNA chemical modifications in eukaryotes and can modulate RNA splicing, degradation, transport and translation with the assistance of m^6^A readers [14–16]. Recent reports uncovered the essential role of m^6^A in bone-related diseases, such as osteoporosis, osteoarthritis, and fracture healing [17–19]. Our previous data elucidated the accelerative effect of N6-adenosine methyltransferase METTL3 on the bone-building ability of bone marrow mesenchymal stem cells (BMSCs) and preosteoblasts [20, 21]. METTL3 modulated osteoblast differentiation and apoptosis in LPS-induced inflammatory environment [21, 22]. However, whether METTL3-mediated m^6^A regulates the development of periodontitis remains unknown. The researches of METTL3 in osteoblast ribosome biogenesis and global protein synthesis are also limited. This study aimed to investigate the specific regulatory role of METTL3 in ribosomal function in LPS-stimulated osteoblasts and explore the effect of METTL3 inhibitor in periodontitis mice.

## Results

### RNA-seq identifies ribosome as METTL3 potential target in LPS-stimulated osteoblasts

**Fig. 1:**
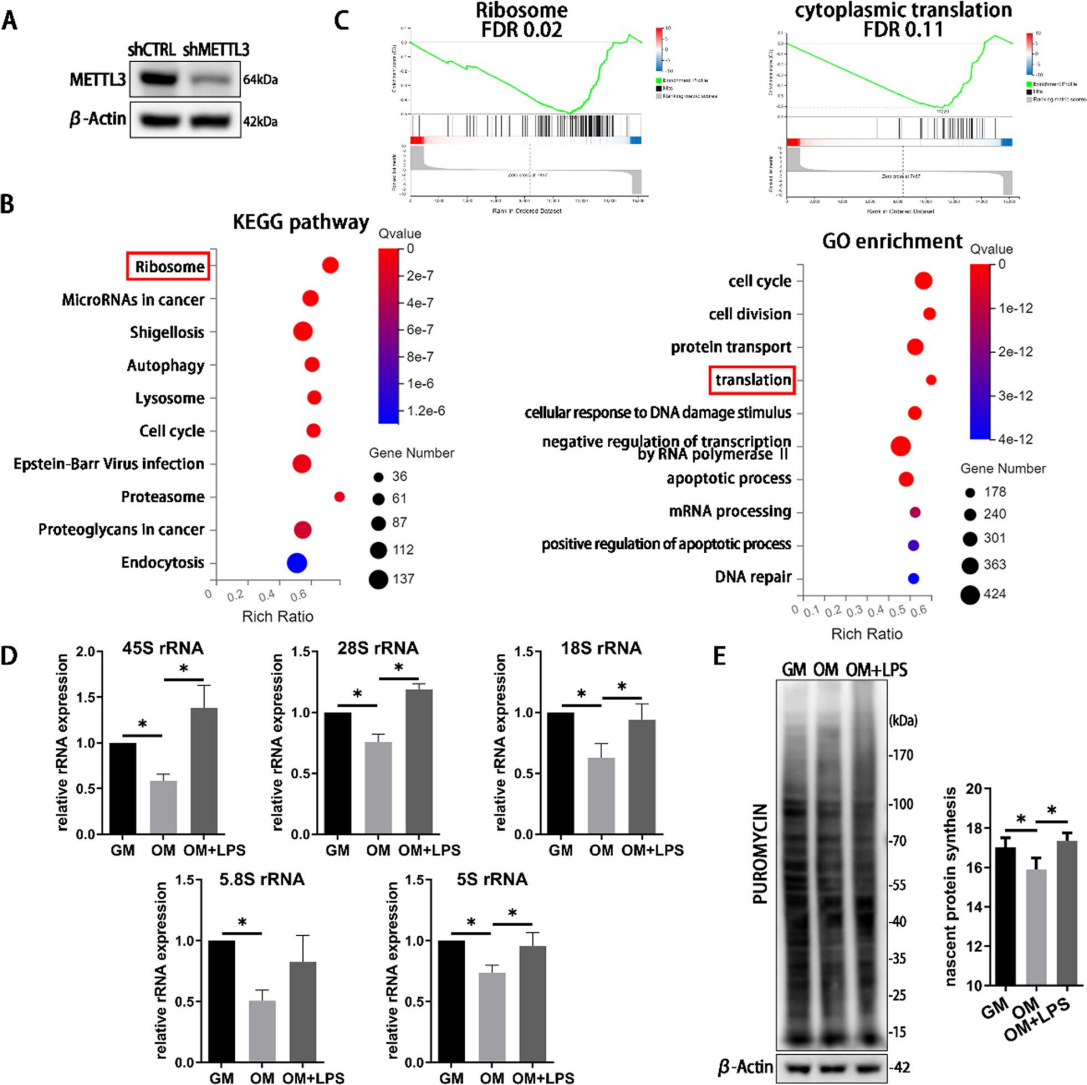
Identified of ribosome as METTL3 target in RNA-seq analysis in LPS-stimulated osteoblasts. **A** The knockdown efficiency was verified by western blotting. **B** KEGG pathway and GO enrichment analysis shown the differentially expressed transcripts between shCTRL and shMETTL3 group. **C** The GSEA enrichment plot of the gene sets. **D** The rRNAs expressions were detected after osteogenic and LPS stimulation 3 days. *n* = 4. GM, growth medium; OM, osteogenic induction medium. **E** The cells were treated with 5 μg/mL puromycin for 40 min. The nascent polypeptides were measured by western blotting. β-Actin was used as internal control. *n* = 3. All data represent the mean ± SD. *P < 0.05

Our previous study has elucidated that METTL3 expression was promoted during osteogenic induction but suppressed in LPS-stimulated osteoblasts. METTL3 depletion inhibited the osteoblast differentiation and promoted inflammatory response upon LPS stimulation [21]. To further investigate the potential mechanism of METTL3 in inflammation-induced osteoblast dysfunction, METTL3 was knocked down by shRNA as our previous study [22]. RNA-seq analysis was performed after METTL3 knockdown (Fig. 1A). KEGG pathway and GO enrichment analysis showed that the differentially expressed genes were enriched in ribosome and translation (Fig. 1B). GSEA analysis exhibited the decline of ribosome and cytoplasmic translation processes (Fig. 1C). Thus, the level of ribosome biogenesis was examined in LPS-stimulated osteoblasts. The expression of rRNAs and nascent protein synthesis decreased after osteogenic induction but increased after LPS stimulation (Fig. 1D, E). Accordingly, the RNA-seq results indicated that METTL3 might have a regulatory role in the process of ribosome biogenesis to support osteoblast activities.

### METTL3 regulates osteoblast ribosome biogenesis and global translation

**Fig. 2:**
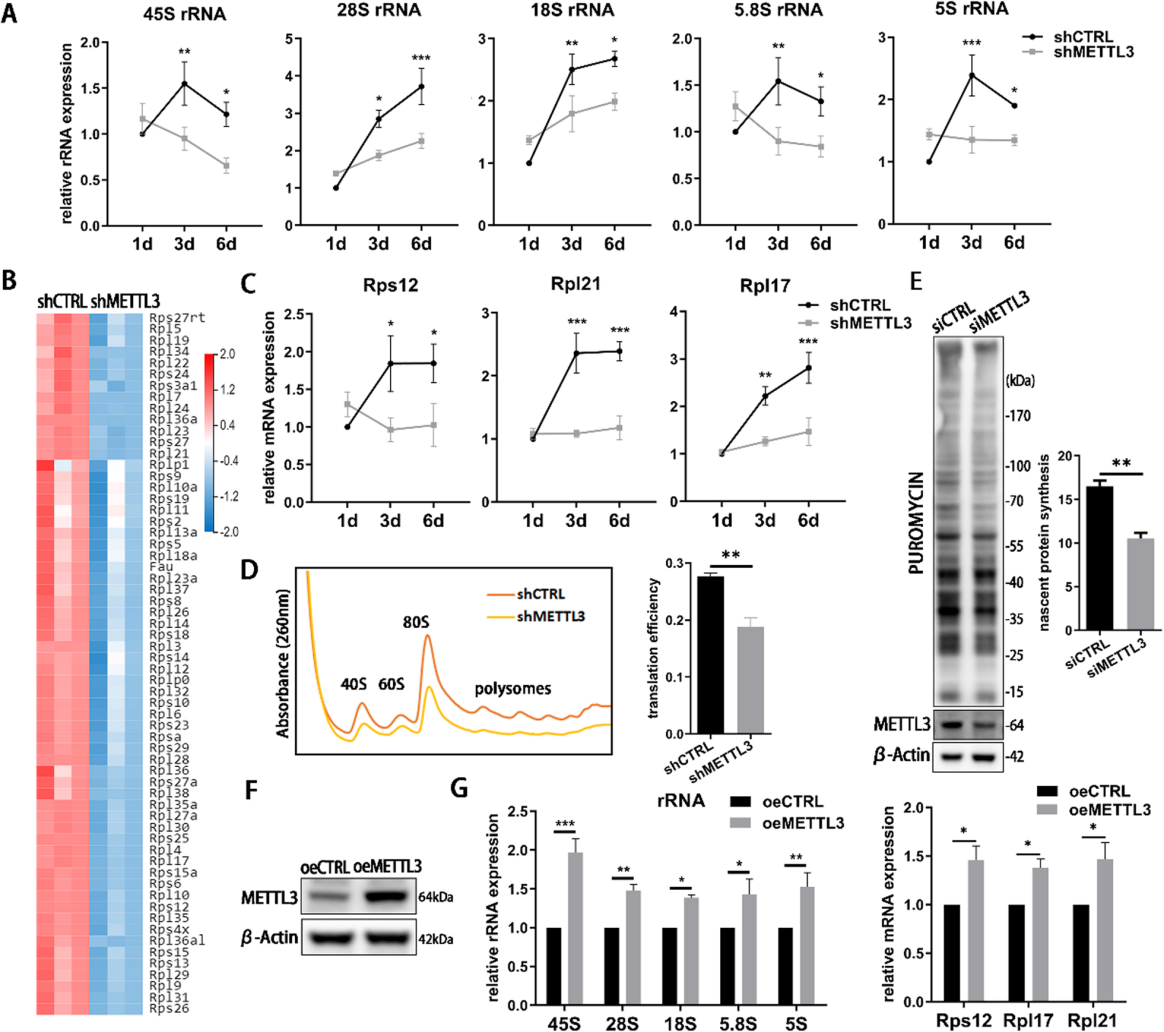
The effect of METTL3 knockdown and overexpression on ribosome biogenesis. **A**, **C** The expression of rRNAs and RPs was detected in METTL3 knockdown cells after LPS and osteogenic induction for 3 days. rRNAs, *n* = 4. RPs, *n* = 3. **B** A heatmap of differentially expressed RP genes. **D** The ribosome fractions were separated and quantitated by absorbance at 260 nm. The translation efficiency was recorded as the ratio between the absorbance of polysomes and not translating monosomes. **E** The protein synthesis rate was measured by puromycin incorporation assay in siMETTL3 cells. *n* = 3. **F** The overexpressing efficiency of METTL3 was tested by western blotting. **G** The expression of rRNAs and RPs was evaluated in METTL3-overexpressed cells. *n* = 3. All data represent the mean ± SD. *P < 0.05; **P < 0.01; ***P < 0.001

To determine the influence of METTL3 in ribosome biogenesis in periodontitis, the ribosomal synthesis and function were measured in LPS-stimulated osteoblasts. Compared with negative control, the expression of pre-rRNA, 28S, 18S, 5.8S and 5S rRNA was reduced after METTL3 inhibition (Fig. 2A). RNA-seq data exhibited the heat map of RPs in Fig. 2B. We chose three significantly expressed RP genes to verify the RNA-seq results and found that the expression of RPs decreased in METTL3 knockdown group (Fig. 2C). METTL3 knockdown also inhibited osteoblast ribosomal subunits and polysomes. The translation efficiency of polysome was reduced in METTL3 knockdown cells (Fig. 2D). To avoid the anti-puromycin effect of virus, we used siRNA to knock down METTL3 and observed that METTL3 depletion decreased osteoblast nascent protein level (Fig. 2E). Conversely, the expression of rRNAs and RPs was upregulated in oeMETTL3 cells (Fig. 2F, G). Surprisingly, the RNA decay of rRNAs showed unchanged after METTL3 knockdown (Additional file 1: Fig. S1A). Altogether, these findings demonstrated that METTL3 acts as a positive regulator of ribosome biogenesis and global translation efficiency in LPS-stimulated osteoblasts.

### METTL3 modulates oxidative phosphorylation and ATP production

**Fig. 3:**
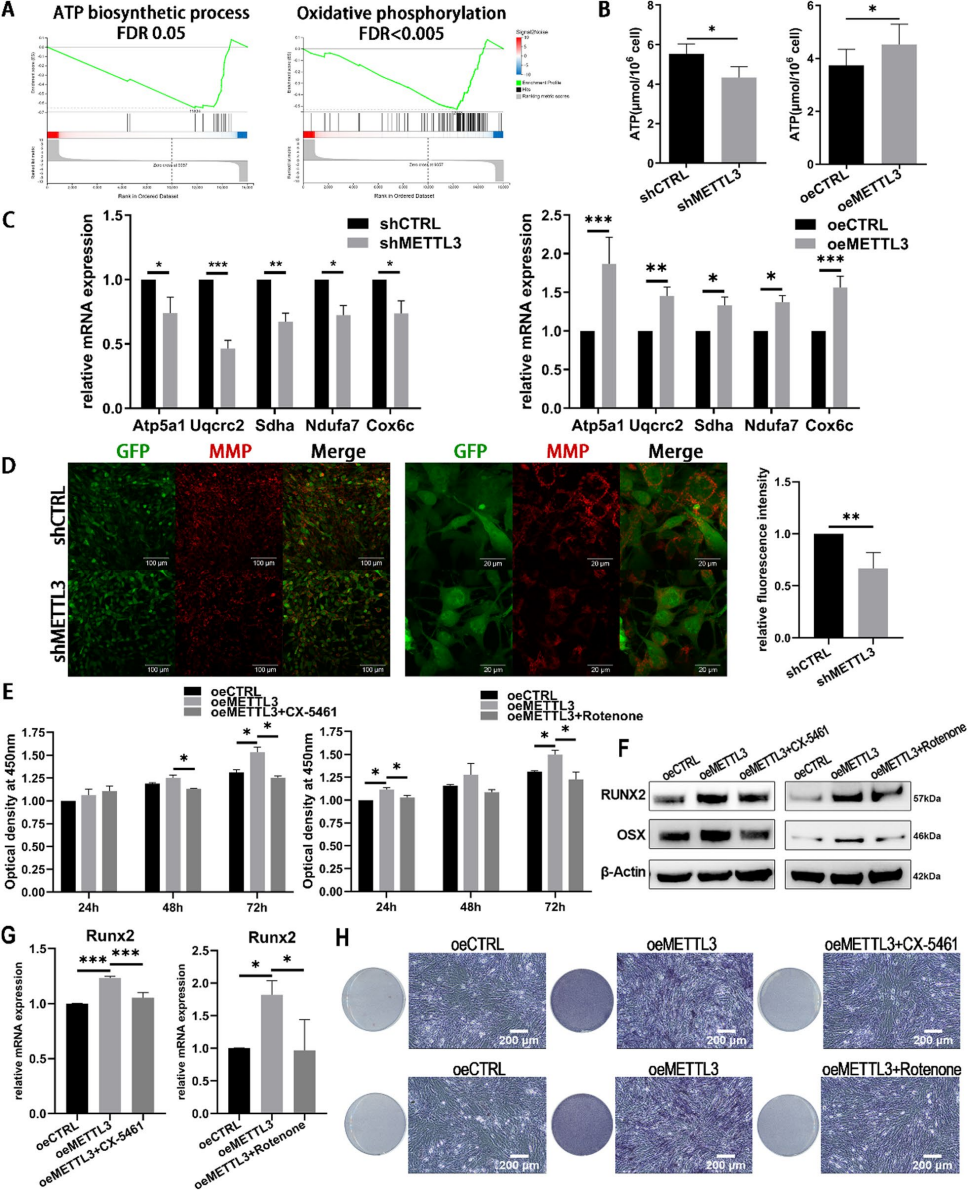
The effect of METTL3 on oxidative phosphorylation and osteoblast differentiation. **A** GSEA analysis found that METTL3 depletion affected the genes in the ATP biosynthetic process and oxidative phosphorylation. **B** The ATP level was assessed in LPS-treated osteoblasts after METTL3 knockdown and overexpression. *n* = 3. **C** The expression of respiratory chain genes was detected at day 3. Knockdown, *n* = 4. Overexpression, *n* = 3. **D** The level of MMP was measured after METTL3 knockdown. *n* = 3. **E**–**H** MC3T3-E1 cells were treated by osteogenic induction medium and LPS with or without 100 nM CX-5461/rotenone. Cell proliferation was analyzed by CCK8 assay (**E**). The expression level of RUNX2 and OSX on day 3 was measured by western blotting (**F**) and qRT-PCR (**G**). The ALP staining was performed on day 7 (**H**). *n* = 3. All data represent the mean ± SD. *P < 0.05; **P < 0.01; ***P < 0.001

Ribosome biogenesis is generally known as the most energy-consuming cellular process, and oxidative phosphorylation is the vital energy producing way [23]. GSEA analysis found that the ATP biosynthetic process and oxidative phosphorylation were significantly downregulated by METTL3 depletion (Fig. 3A). To explore the effect of METTL3 on energy metabolic process, ATP production and mitochondrial respiration were tested. Knocking down METTL3 reduced ATP level and respiratory chain genes expression, while overexpressing METTL3 promoted them (Fig. 3B, C). Moreover, METTL3 inhibition restrained MMP and improved the release of ROS (Fig. 3D, Additional file 1: Fig. S2A, B). These data confirmed that METTL3 modulates oxidative phosphorylation and ATP generation in LPS-treated osteoblasts.

To further validate the role of ribosome biogenesis and respiratory chain in METTL3-regulated osteoblast activities, cells were treated with CX-5461 or rotenone. CX-5461 is a potent bioavailable inhibitor of RNA Pol I-mediated rRNA synthesis. Rotenone is a mitochondrial electron transport chain complex I inhibitor and can suppress ATP production. As shown in Fig. 3E, overexpressing METTL3 enhanced the proliferation of osteoblasts, while CX-5461 restrained the cell proliferation. Rotenone also inhibited the cell activity in METTL3-overexpressed osteoblasts. The expression of RUNX2 and OSX increased in oeMETTL3 cells but decreased after CX-5461 or rotenone treatment (Fig. 3F, G). The ALP activity showed the same trend (Fig. 3H). These results indicated that METTL3 regulates ribosome biogenesis and mitochondrial respiration to affect osteoblast proliferation and differentiation.

### METTL3 activates Wnt/β-catenin/c-Myc signaling to promote ribosome biogenesis and ATP production

**Fig. 4:**
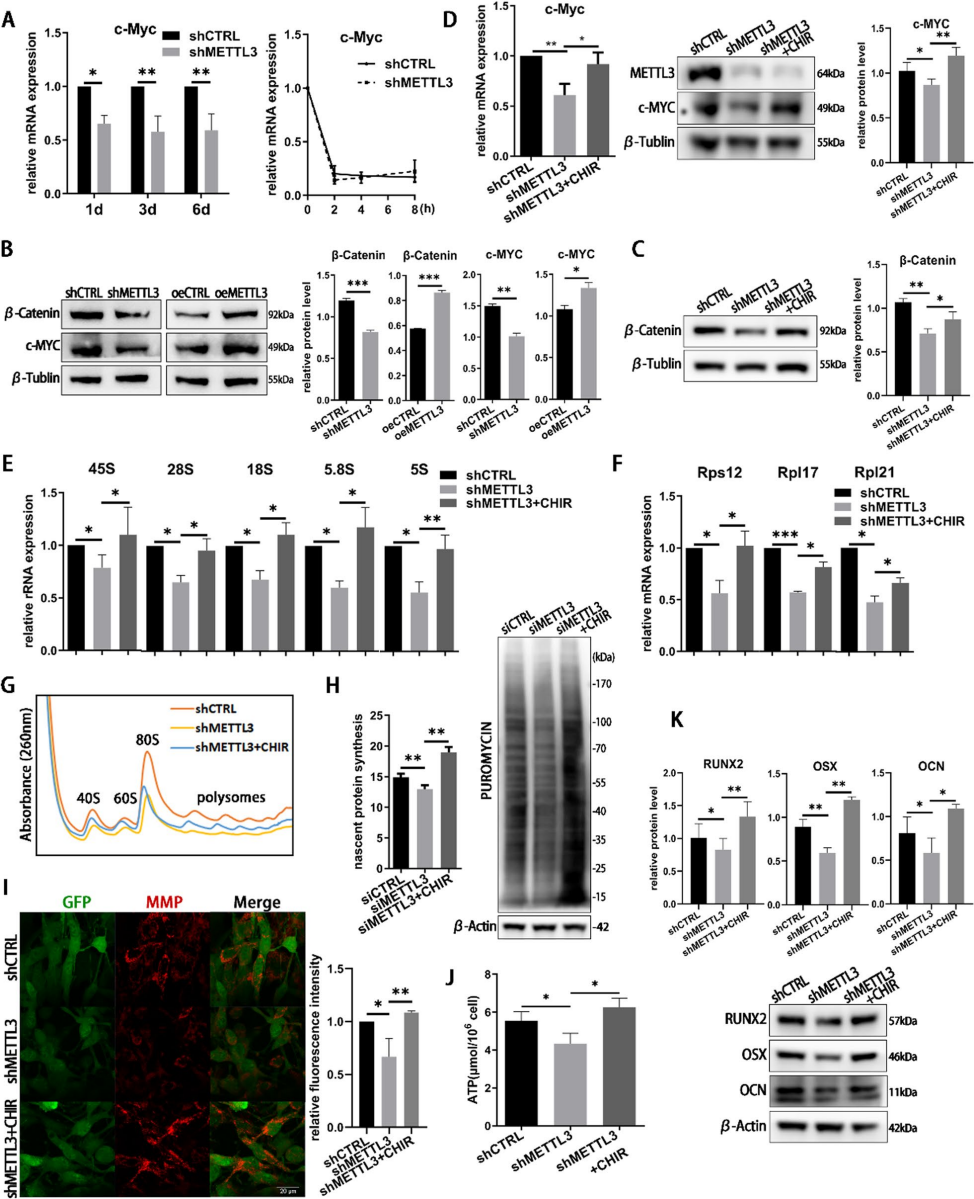
The role of Wnt/β-catenin/c-Myc signaling in METTL3-depletion cells. **A** The expression and mRNA degradation of c-Myc were tested by qRT-PCR. *n* = 3. **B** The protein level of β-catenin and c-Myc on day 3 was measured by western blotting. **C**–**F** The cells were treated with or without CHIR for 3 days. The expression of β-catenin, c-Myc, rRNAs and RPs was evaluated by qRT-PCR and western blotting. c-Myc, rRNAs, *n* = 4. RPs, *n* = 3. **G**, **H** The ribosomal level and neo protein synthesis rate were measured on day 3. *n* = 3. **I**, **J** The ATP level and MMP was detected on day 3. *n* = 3. (K) The osteogenic markers RUNX2, OSX, and OCN were measured by western blotting. All data represent the mean ± SD. *P < 0.05; **P < 0.01; ***P < 0.001

To explore the regulatory pathway of METTL3 in ribosome biogenesis and ATP production, we evaluated the nucleolus and the signaling pathways linked with the ribosomal process. The AgNOR staining and immunocytochemistry of NPM and Fibrillarin showed that nucleolar morphology and number had no significant changes in METTL3 knockdown cells (Additional file 1: Fig. S3A–C). The unchanged p53 pathway and activated mTOR-Akt pathway did not accord with the changes of ribosome biogenesis as reported (Additional file 1: Fig. S3D, E). Given the findings that Wnt/c-Myc signaling regulates the process of ribosome biogenesis [24, 25], the level of c-Myc was assessed. The results indicated that METTL3 depletion decreased the expression of c-Myc and METTL3 overexpression resulted in the reverse effect (Fig. 4A, B). Nevertheless, the mRNA stability of c-Myc was unchanged after METTL3 knockdown (Fig. 4A). The level of β-catenin pathway, the upstream of c-Myc, was then measured and the results showed that β-catenin was suppressed in shMETTL3 cells and enhanced in oeMETTL3 cells (Fig. 4B).

To further validate the role of canonical Wnt/β-catenin/c-Myc pathway in osteoblast ribosome biogenesis, cells were treated with the pathway activator CHIR-99021 HCl (CHIR). CHIR activated the Wnt/β-catenin pathway and rescued the expression level of c-Myc with a marginal effect on METTL3 expression (Fig. 4C, D). The inhibition of rRNA, RPs, ribosome and global translation in METTL3 knockdown cells was reversed after using CHIR (Fig. 4E–H). CHIR treatment also enhanced the ATP production and mitochondrial membrane potential in shMETTL3 cells (Fig. 4I, J). In agreement with our previous research that METTL3 depletion significantly inhibited osteoblast differentiation [22], the protein levels of osteoblast marker Runx2, Osterix (OSX), and osteocalcin (OCN) were inhibited in METTL3 knockdown cells, which could be rescued by CHIR administration (Fig. 4K). Consequently, we concluded that METTL3 activates Wnt/β-catenin/c-Myc to promote ribosome biogenesis and ATP production in LPS-treated osteoblasts.

### METTL3 regulates Dkk3 and Sostdc1 mRNA decay in a YTHDF2-dependent way

**Fig. 5:**
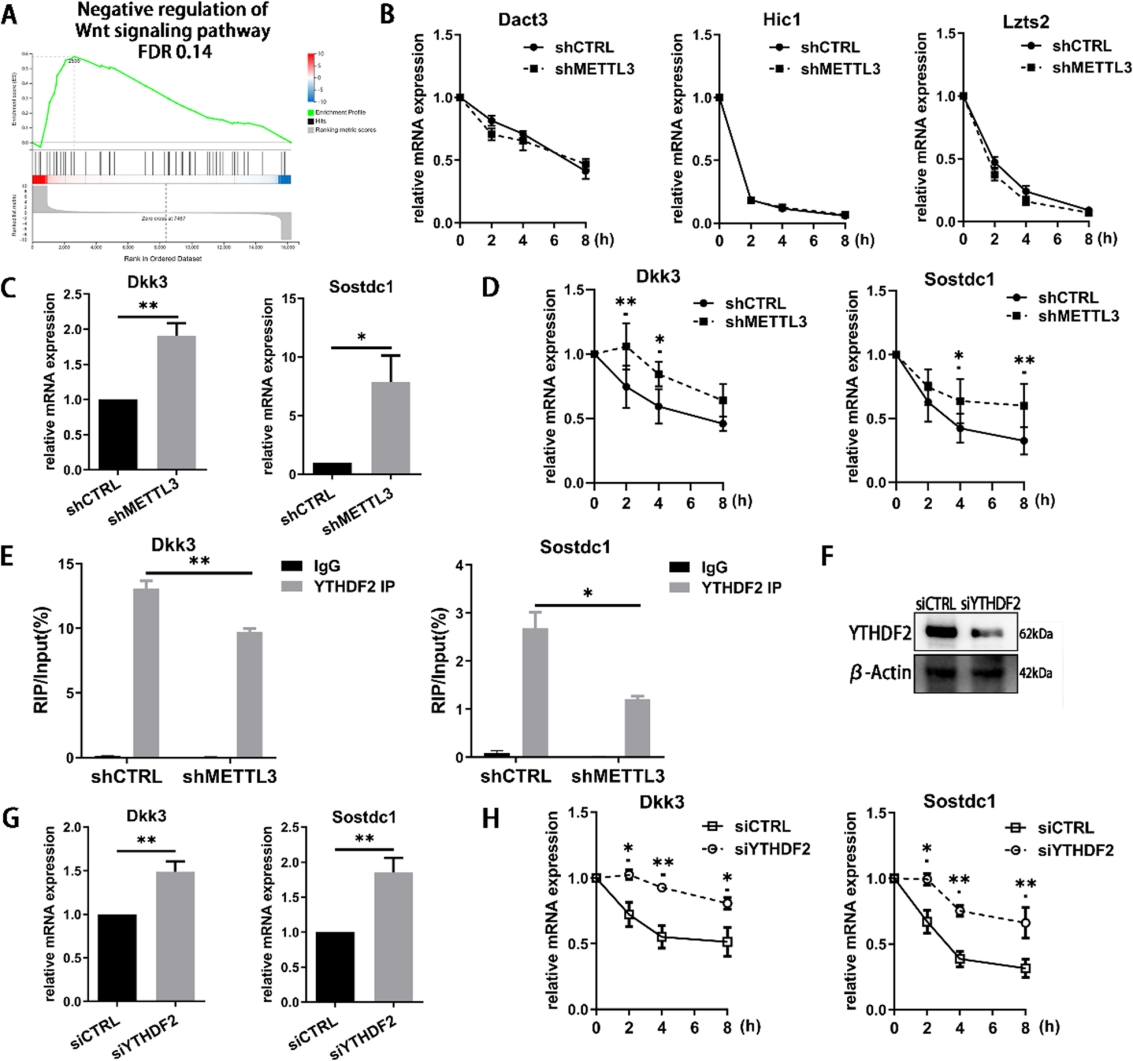
The effect of METTL3 and YTHDF2 on the mRNA stability of Dkk3 and Sostdc1 in Wnt/β-catenin signaling. **A** GSEA analysis identified the negative regulation of Wnt signaling pathway. **B**, **D**, **H** The METTL3/YTHDF2-kncokdown cells were stimulated with LPS and osteogenic induction medium for 64 h and then treated with 5 μg/mL ActD for 0–8 h. The mRNA expression of Dact3, Hic1, Lzts2, Dkk3 and Sostdc1 was measured by qRT-PCR. *n* = 3. **C**, **G** The expression of Dkk3 and Sostdc1 was examined by qRT-PCR during 3-day LPS-stimulated osteoblast differentiation. *n* = 4. **E** The binding between YTHDF2 and Dkk3 or Sostdc1 was determined by RIP assay. *n* = 3. **F** The knockdown efficiency was verified by western blotting. The results are shown as the mean ± SD. *P < 0.05; **P < 0.01

The activation of canonical β-catenin-dependent pathway is regulated by a number of secreted extracellular antagonists. GSEA analysis indicated that the negative regulation of Wnt signaling pathway was increased in shMETTL3 cells (Fig. 5A). To elucidate how METTL3 affected these negative regulators, the mRNA decay of the significantly expressed genes was tested. The mRNA degradation of Dact3, Hic1 and Lzts2 performed unchanged (Fig. 5B), while Dkk3 and Sostdc1 had significant differences. METTL3 depletion enhanced Dkk3 and Sostdc1 expression and RNA stability (Fig. 5C, D). Nevertheless, the Wnt pathway activator CHIR did not reverse the increased expression of Dkk3 and Sostdc1 in METTL3-deficent cells (Additional file 1: Fig. S4A, B).

METTL3 generally exerts its effect through selective recognition by m^6^A reader. YTHDF2 is the main m^6^A reader that is identified to recognize and affect the stability of the m^6^A-modified mRNA [26]. Our results showed that Dkk3 and Sostdc1 could bind with YTHDF2 and the binding was inhibited by METTL3 knockdown (Fig. 5E). Knocking down YTHDF2 increased the mRNA stability and level of Dkk3 and Sostdc1 (Fig. 5F–H). Thus, METTL3 promoted Dkk3 and Sostdc1 mRNA degradation via a YTHDF2-dependent manner.

### METTL3 inhibitor SAH facilitates the bone loss and inflammatory response in periodontitis

**Fig. 6:**
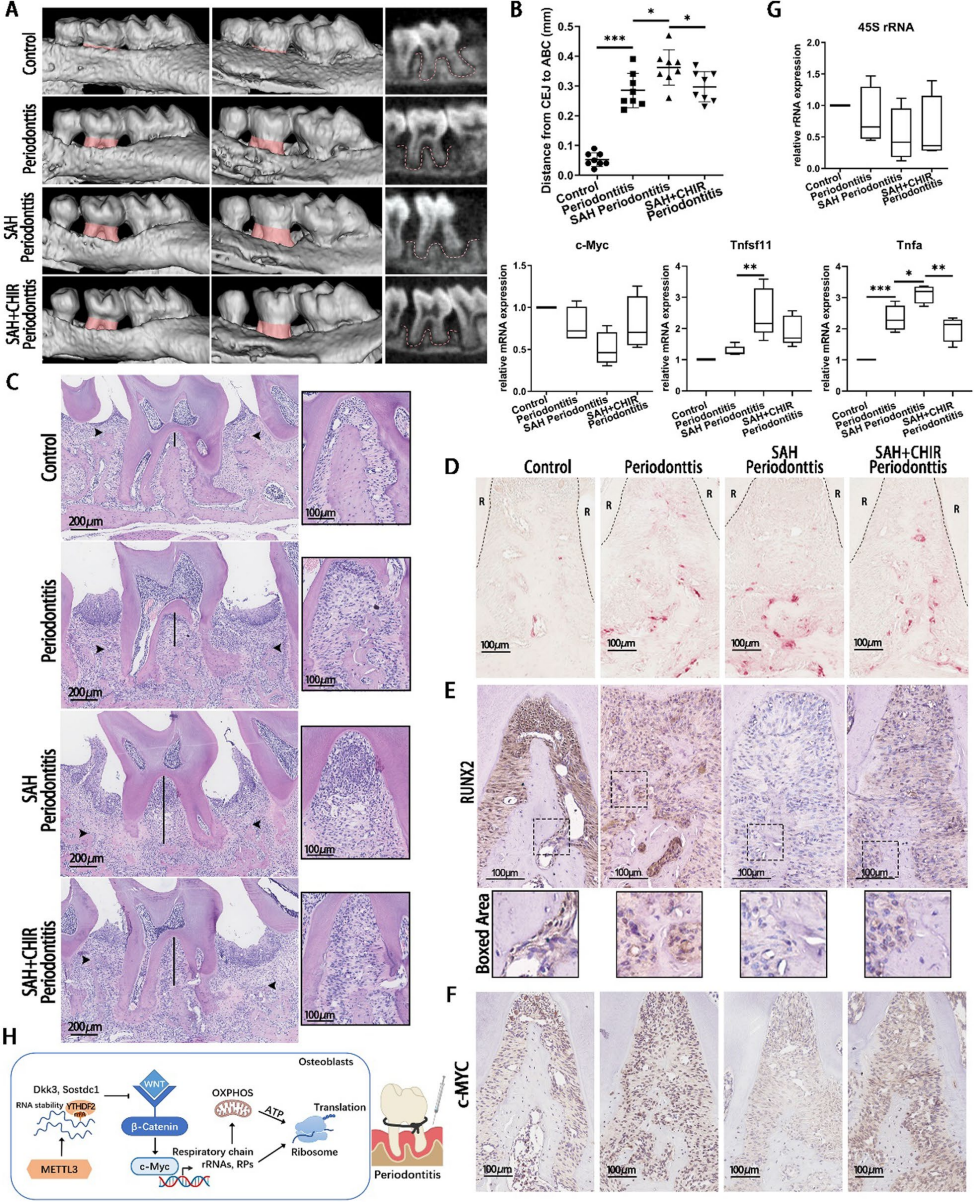
The effect of SAH and CHIR in osteoblasts and periodontitis mice. **A**, **B** Representative micro-CT reconstruction images of the maxillae. Red area showed the CEJ-ABC distance. Red lines showed the alveolar bone. CEJ, cementum enamel junction; ABC, alveolar bone crest. **C** H&E staining images of the periodontal tissues. Black arrows point to ABC. Black lines represent the bone loss in root furcation. **D** TRAP staining images in root furcation. R, root. **E**, **F** IHC staining of RUNX2 and c-MYC in the periodontal ligament and alveolar bone. **G** The expression of c-Myc, 45S rRNA, RANKL(Tnfsf11) and TNF-α(Tnfa) of periodontal tissues was detected by qRT-PCR. *n* = 3. **H** Schematic diagram illustrating the mechanism of METTL3 regulating osteoblast ribosome biogenesis and oxidative phosphorylation via Wnt/β-catenin/c-Myc signaling in periodontitis. All data represent the mean ± SD. *P < 0.05; **P < 0.01; ***P < 0.001

In order to determine the effect of METTL3 in periodontitis, a known methyltransferase inhibitor SAH was used to treat LPS-stimulated osteoblasts in vitro and injected into ligature-induced periodontitis model in *vivo*. SAH suppressed the protein levels of β-catenin, c-MYC, and RUNX2, whereas CHIR rescued the effect of SAH in osteoblasts (Additional file 1: Fig. S5A). In murine periodontitis, alveolar bone destruction, collagen fibers destruction, inflammatory cell infiltration and osteoclastogenesis increased in SAH group, compared with those in periodontitis group (Fig. 6A–D, Additional file 1: Fig. S5B). The RUNX2 positive cells in the periodontal ligament and alveolar bone decreased in periodontitis group and was further reduced in SAH group (Fig. 6E). The expression of c-Myc and 45S rRNA showed a decreasing trend, while the expression of RANKL and TNF-α increased after injecting SAH (Fig. 6F, G). All these effects of SAH were reversed by the Wnt pathway activator CHIR (Fig. 6A–G). The alveolar bone erosion, collagen fibers destruction, and inflammatory response were attenuated after CHIR treatment (Fig. 6A–D, Additional file 1: Fig. S5B). These results collectively suggested that the alveolar bone loss and inflammation in periodontitis were worsened by METTL3 inhibitor SAH and this deteriorative effect was partially rescued by CHIR.

## Discussion

Periodontitis is a common infectious disease that affect bone, gingiva, and periodontal ligament, involving complex dynamic interactions among bacterial pathogens and host immune responses. Periodontal pathogen inhibits the function of osteoblasts, thereby disturbing the strict synchronization of bone resorption and formation in periodontal tissue [27, 28]. Recent studies have demonstrated that osteoblast activity is controlled by m^6^A methylation, the most ubiquitous mRNA modification in eukaryotes [29, 30]. Our previous research demonstrated that the levels of m^6^A methyltransferase METTL3 were promoted during osteogenic induction but suppressed upon LPS treatment. METTL3 promoted the osteoblast differentiation and inhibited inflammatory response in both osteogenic induction and LPS-induced environments [21, 22].

In order to investigate the underlying mechanism of METTL3-mediated osteoblast activity in periodontitis, RNA-seq was performed in the present study and data showed that the genes of ribosome and cytoplasmic translation were downregulated in METTL3 knockdown osteoblasts after LPS treatment. The ribosome is a multi-unit complex that translates mRNA into protein. Ribosome biogenesis is the process that generates ribosomes and plays an essential role in regulating cellular homeostasis [31]. Importantly, changes in ribosome biogenesis, both physiologically and pathologically, are now known to be critically important for cell fate determination [8, 32]. Mutations in ribosomal proteins and regulators of ribosome biogenesis cause ribosomopathies with skeletal defects, such as Treacher Collins syndrome, indicating the critical role of ribosome biogenesis in bone development [33, 34]. In the present study, the level of rRNAs and global translation decreased during osteoblast differentiation and increased in LPS-induced inflammation, implying that ribosome biogenesis might be pivotal for osteoblast biological behaviors. Recently, METTL3 was proved to sustain aberrant ribosome level and translation to regulate the cell proliferation in chronic myeloid leukemia [35]. Our research found that the expression of rRNAs and RPs, ribosomal amount, and translational rate were inhibited after METTL3 depletion and enhanced after METTL3 overexpression under LPS-induced condition. These results uncovered the promoting role of METTL3 in ribosome biogenesis in LPS-treated osteoblasts.

Ribosome biogenesis is the most energy-demanding process in eukaryotic cells, consuming a large portion of energy. It requires extensive regulation and coordination with other cellular pathways [23, 36]. The GSEA analysis of RNA-seq identified the impairment of ATP biosynthetic process and oxidative phosphorylation in METTL3 knockdown cells in this study. Oxidative phosphorylation is the final common pathways for the generation of ATP needed to fuel bone formation [37, 38]. Our data confirmed that METTL3 knockdown prevented the ATP production and the respiratory chain gene expression, whereas METTL3 overexpression exhibited the reverse effect. METTL3 knockdown also reduced MMP level and increased ROS release. The ribosome biogenesis inhibitor CX-5461 and the respiratory chain inhibitor rotenone both showed suppressive impacts on the osteoblast proliferation and differentiation in METTL3-overexpressed cells. Therefore, METTL3 mediated oxidative phosphorylation and ATP production in LPS-stimulated osteoblasts. METTL3 promoted ribosome biogenesis and mitochondrial respiration to affect osteoblast proliferation and differentiation.

The nucleolus is the hub for ribosome biogenesis, in which the initial and speed-limiting step takes place. Ribosomal stress, also called nucleolar stress, refers to the collapse of nucleolar gross architecture and function induced by abnormal conditions [39, 40]. In the present study, no significant change of the nucleolar number and morphology was found in METTL3 knockdown osteoblasts. The RNA stability of rRNAs also performed unchanged after knocking down METTL3, indicating that the regulation of METTL3 on rRNA metabolism was limited. Multiple signal transduction events have been reported to control ribosome biogenesis and protein synthesis, such as Wnt/c-Myc, Mdm2-p53, and PI3K-Akt-mTOR pathways [41–43]. Surprisingly, the p53 pathway remained unchanged and the mTOR-Akt pathway was activated in this study, which were at odds with the trend of ribosome biogenesis in osteoblasts. It is generally acknowledged that Wnt signaling participates in osteoblast differentiation and metabolism. When the canonical pathway is activated, accumulated β-catenin translocates into the nucleus and induce the transcription of target genes, such as c-Myc [44]. Our results indicated that METTL3 activated the Wnt/β-catenin/c-Myc pathway in LPS-stimulated osteoblasts. The expression of c-Myc and β-catenin decreased after METTL3 knockdown and increased following METTL3 overexpression, though the mRNA stability of c-Myc was unchanged. Wnt pathway activator CHIR was then used to rescue Wnt/β-catenin/c-Myc pathway. The results showed that CHIR restored the expression levels of c-Myc and β-catenin in METTL3-depletion cells, with little impact on the METTL3 expression. Further, the ribosome biogenesis and global translation were enhanced, and ATP production and oxidative phosphorylation were rescued by CHIR. The protein levels of the osteogenic markers were also retrieved after CHIR treatment. These findings demonstrated that METTL3 mediated Wnt/β-catenin/c-Myc axis to promote osteoblast ATP production, ribosome biogenesis and osteoblast differentiation in LPS-stimulated condition.

The activation of canonical β-catenin-dependent pathway is determined by numerous factors. The Dickkopf (Dkk) family and Sostdc1 are secreted proteins that can competitively bind to the extracellular domains of Lrp5/6 and disturb their interaction with Wnt proteins [45, 46]. In the present study, GSEA analysis showed that METTL3 inhibition elevated the expression of Wnt signaling pathway negative regulators. To further explore the mechanism of Wnt/β-catenin/c-Myc pathway in METTL3-mediated osteoblasts, the differently expressed regulators in this gene set were assessed. The results showed that the mRNA expression and stability of Dkk3 and Sostdc1 increased after METTL3 knockdown, indicating a possible regulatory way of METTL3 in Wnt pathway. The m^6^A sites on Dkk3 and Sostdc1 mRNA were analyzed by SRAMP (http://www.cuilab.cn/sramp) and we found that both mRNAs have m^6^A sites, suggesting that the regulation of METTL3 on the mRNA decay of Dkk3 and Sostdc1 might be m^6^A-dependent [47]. The molecular and biological functions involving m^6^A are mostly mediated by m^6^A-recognizing RNA binding proteins (also known as m^6^A readers), such as YTH domain-containing proteins. Recent reports outlining the mechanism behind the decay of m^6^A-modified mRNAs indicate that YTHDF2 is the major decay-inducing reader protein via recruiting the CCR4-NOT deadenylation complex or by an HRSP12-dependent endoribonucleolytic-cleavage pathway [15, 48]. Recent evidence showed that METTL3 promotes cholangiocarcinoma progression by YTHDF2-mediated IFIT2 mRNA degradation [49]. METTL3 facilitates bladder cancer growth and metastasis by suppressing RRAS expression in a YTHDF2‑dependent manner [50]. To illuminate if YTHDF2 recognized the mRNA of Dkk3 and Sostdc1 and modulated the mRNA decay, RIP-PCR and RNA stability assay were performed. The results indicated that the binding of Dkk3, Sostdc1 mRNA and YTHDF2 protein was regulated by METTL3. YTHDF2 knockdown reduced the mRNA degradation of Dkk3 and Sostdc1 and accelerated their expression. Collectively, METTL3 promoted the mRNA decay of Wnt signaling antagonists Dkk3 and Sostdc1 via a YTHDF2-dependent manner in osteoblasts.

SAH is an amino acid derivative that has strong inhibitory effect on METTL3 activity with an IC_50_ value of 0.9 ± 0.1 µM [51]. To further confirm the effect of METTL3, SAH was used to treat LPS-stimulated osteoblasts. The expression of β-catenin, c-MYC, and RUNX2 in osteoblasts was suppressed by SAH and reversed by CHIR [4]. To evaluate the effect of METTL3 in vivo, a ligature-induced periodontitis model was built in mice and SAH was injected into the palatal mucosa of maxillary second molars. The histological results indicated that SAH injection deteriorated the alveolar bone destruction and inflammatory response and inhibited the bone regeneration, which were partially rescued by CHIR treatment. The expression of RANKL and TNF-α displayed the same trend with bone resorption and inflammatory response. The RUNX2 positive cells decreased after SAH injection and increased after CHIR treatment. IHC staining showed that the protein expression of c-MYC in periodontal tissue decreased in SAH group and increased in SAH + CHIR group. A similar trend was observed for 45S rRNA level. Accordingly, METTL3 inhibitor promoted local inflammatory osteolysis in periodontitis mice, while Wnt signaling activator CHIR partially reversed the effect of SAH.

## Conclusions

This study aimed to clarify the role of m^6^A methyltransferase METTL3 in osteoblast biology process in periodontitis and explore its potential mechanism. METTL3 activated Wnt/β-catenin/c-Myc axis to facilitate ribosome biogenesis and translation rate, which influenced osteoblast function in periodontitis. Mechanistically, METTL3 inhibited Dkk3 and Sostdc1mRNA stability in a YTHDF2-dependent manner to promote Wnt signaling pathway. These findings highlight the importance of METTL3-meditated Wnt/β-catenin/c-Myc signaling in osteoblast activity and provide a promising therapeutic target for periodontal disease.

## Methods

### Cell culture and treatment

MC3T3-E1 cells were cultured and osteogenic induced according to our previous study [21]. *Escherichia coli* LPS (Sigma, St. Louis, MO, USA) of 1 μg/mL was added into the osteogenic medium. CHIR-99021 HCl (CHIR) (Selleck, Shanghai, China) was used at 3 μM for indicated times.

### Cell transfection

Lentiviruses were all purchased from Shanghai Genechem company. The shRNA sequences against METTL3 were CGTCAGTATCTTGGGCAAATT, and non-specific control shRNA were TTCTCCGAACGTGTCACGT. METTL3 overexpressing lentivirus contained the full-length sequences of METTL3. The lentiviruses were transduced into cells with a multiplicity of infection of 80. Transfected cells were maintained with puromycin (Sigma) of 6 µg/mL for more than 3 d. The siRNA against METTL3, YTHDF2 or negative control was transfected as previous description [21].

### RNA sequencing (RNA-seq)

Approximately 20 ng of Poly(A) RNA was purified from the shCTRL and shMETTL3 cells after stimulation 72 h with two biological replicates in each group. After reverse transcribing into cDNA, samples were sequenced by BGI-SEQ500 platform (BGI, Shenzhen, China). The expression was quantified as reads per kilobase per million reads (RPKM). Differentially expressed genes were identified by fold change >|1| and *P* < 0.05. Gene Ontology (GO) and Kyoto Encyclopedia of Genes and Genomes (KEGG) analyses were performed with the BGI data analysis platform and bioinformatics.

### Real-time quantitative polymerase chain reaction (qRT-PCR)

Total RNA was extracted by RNAzol (MRC, OH, USA) and converted into cDNA by the Takara PrimeScriptTM RT kit. RT-PCR was performed with Roche SYBR Green I Master Mix using QuantStudio 5. The primer sequences are listed in Additional file 1: Table S1. β-Actin was used as a normalization control.

### Western blotting

Total proteins were extracted by RIPA lysis buffer. Equal quality proteins were separated by 10% SDS-PAGE and transferred onto the PVDF membranes. The membranes were blocked followed by incubation with primary antibodies against RUNX2, OSX, OCN, c-MYC (Affinity Biosciences, Cincinnati, OH, USA), METTL3, YTHDF2 (Proteintech, Chicago, IL, USA), PUROMYCIN (Kerafast, Boston, MA, USA), p-p53, p53, p-AKT, AKT, p-mTOR, β-catenin, β-actin, β-tublin, and vinculin (CST, Boston, MA, USA) and secondary antibodies (CST). Target protein bands were visualized by ECL (Millipore) and quantified by ImageJ.

### RNA immunoprecipitation (RIP)-qPCR analysis

For RNA immunoprecipitation, the Magna RIPTM kit (Millipore, Billerica, MA, USA) was used in accordance with the manufacture instructions. 2 × 10^7^ cells were lysed and immunoprecipitated with YTHDF2 antibody or IgG overnight. The bound RNA was then assessed by qRT-PCR.

### Mitochondrial membrane potential (MMP) analysis and reactive oxygen species (ROS) analysis

To assess the level of MMP, cells were incubated with 100 nM Mito-Tracker Red CMXRos (Beyotime) at 37 °C for 20 min. The MMP was visualized by Laser Scanning Microscope 780 and quantified by ImageJ. For assess the level of ROS, cells were incubated with 40 μM DHE (Vigorous, Beijing, China) for 30 min. The fluorescence intensity was examined by Laser Scanning Microscope 780 and quantified using Flow cytometry (BD LSRFortessa, USA).

### ATP analysis

ATP level was tested by ATP assay kit (Beyotime) in accordance with the manufacture instructions. Cells were lysed, mixed with working buffer, and measured by a luminometer (Biotek, Winooski, VT, USA).

### AgNOR and immunofluorescence staining

For measuring nucleolus number, cells were stained by AgNOR kit (Leagene Biotechnology, Beijing, China) in accordance with the experimental instructions. For assay nucleolar stress, cells were fixed, permeated, and blocked by 5% goat serum. Cells were incubated with primary antibody against NPM (Affinity Biosciences) or Fibrillarin (CST) overnight, and then incubated with secondary antibody, DyLight 649 (EarthOx, Burlingame, CA, USA) and DAPI staining solution (Beyotime, Shanghai, China). The fluorescence was analyzed by Laser Scanning Microscope 780 (Zeiss, German).

### Polysome profiling

The cells were incubated with 100 μg/mL cycloheximide for 20 min. 4 × 10^6^ cells were collected with 500 μl of lysis buffer (Additional file 1: Table S2). Supernatants were then loaded on 5–50% linear sucrose gradients and centrifuged using SW41Ti rotors (Beckman) at 36,000 rpm for 3 h. The polysome profiles were recorded and fractionated with the gradient fractionator (Biocomp, CA, USA).

### Mouse model of periodontitis

All the experiment protocols were performed by the Animal Care and Use Committee of Sun Yat-sen University. Male C57BL/6 mice aged 8 weeks were randomly divided into 4 groups (6 mice each group): control group, periodontitis group, SAH (Selleck) periodontitis group, SAH + CHIR periodontitis group. The periodontitis models were established by ligating silk on bilateral maxillary second molars. SAH and CHIR were dissolved by the solvent (5% DMSO, 5% Tween-80, 40% PEG300, and 50% saline). The solvent (8 μl), SAH (10 μg), and CHIR (6 μg) were locally injected into bilateral palatal mucosa of maxillary second molars every 2 days. After 12-day ligature insult, all animals were killed. The maxillae were collected and hemisected (*n* = 12 each group) for subsequent experiments.

### Micro-computed tomography (micro-CT)

The alveolar bones were fixed in 4% paraformaldehyde for 24 h. A micro-CT scanner (µCT 50; SCANCO Medical AG, Basserdorf, Switzerland) was used for bone scanning and three-dimensional reconstruction. The scanning parameters were as follows: resolution, 20 µm; source voltage, 70 kV; current, 114 µA; aluminum filter, 0.5 mm; exposure time, 300 ms.

### Histological analysis and immunohistochemistry (IHC)

The fixed maxillae were decalcified in 10% EDTA for 1 month. Sections were processed by H&E and TRAP staining. For IHC, sections were blocked 5% BSA after antigen retrieval and stained using primary antibody overnight at 4 °C. The immunoactivity was detected by an HRP‐streptavidin detection system.

### Statistical analysis

Data are expressed as mean ± standard deviation (SD) of at least triplicate experiments independently. Comparisons were analyzed by Student’s t test for two groups or ANOVA for multiple groups (GraphPad, San Diego, CA, USA). The statistically significant level was set at *P* < 0.05.

### Supplementary Information

**Additional file 1: Figure S1.** The effect of METTL3 on the rRNA stability. **A** The rRNA expression in METTL3-kncokdown cells after treating with 5 μg/mL ActD for 0–8 h was measured by qRT-PCR. *n* = 3. **Figure S2.** **A**, **B** The level of ROS was measured after METTL3 knockdown. *n* = 3. All data represent the mean ± SD. **Figure S3.** The effect of METTL3 knockdown on nucleolus, mTOR-Akt, and p53 pathway. **A**, **B** The nucleolar morphology of osteoblasts under LPS and osteogenic induction 3 days was assessed by immunocytochemistry. 20 nM actinomycin D (ActD) was the positive control of nucleolar stress. **C** The nucleolar number was detected by AgNOR staining. **D**, **E** The activation of p53 and AKT-mTOR signaling were examined by western blotting. *P < 0.05; **P < 0.01; ***P < 0.001. **Figure S4.** The effect of CHIR on the expression of Dkk3 and Sostdc1 in METTL3 knockdown cells. **A**, **B** The shCTRL and shMETTL3 cells were stimulated by LPS and osteogenic induction medium with or without CHIR. The mRNA expression of Dkk3 and Sostdc1 was detected by RT-qPCR. *n* = 3. All data represent the mean ± SD. *P < 0.05; ***P < 0.001. **Figure S5.** The effect of SAH and CHIR in periodontitis mice. **A** The proteins were evaluated in LPS-stimulated cells after stimulating with 5 μM SAH and 3 μM CHIR for 3 days. **B** Masson staining images of the periodontium. All data represent the mean ± SD. *P < 0.05; **P < 0.01. **Table S1.** Primer sequences for qRT-PCR. **Table S2.** Polysome profiling buffer.

## Abbreviations

m^6^A: *N*^6^-Methyladenosine
METTL3: Methyltransferase-like protein 3
rRNA: Ribosomal RNA
RP: Ribosomal protein
LPS: Lipopolysaccharide
SAH: *S*-Adenosyl-*L*-homocysteine

## References

[1] Hajishengallis G, Chavakis T (2021). Local and systemic mechanisms linking periodontal disease and inflammatory comorbidities. Nat Rev Immunol.

[2] Hajishengallis G (2014). Immunomicrobial pathogenesis of periodontitis: keystones, pathobionts, and host response. Trends Immunol.

[3] Jain S, Darveau RP (2000). Contribution of *Porphyromonas gingivalis* lipopolysaccharide to periodontitis. Periodontol.

[4] Hathaway Schrader JD, Novince CM (2021). Maintaining homeostatic control of periodontal bone tissue. Periodontology 2000.

[5] Saint-Pastou TC, Gasque P (2017). Bone responses in health and infectious diseases: a focus on osteoblasts. J Infect.

[6] Bianco C, Mohr I (2019). Ribosome biogenesis restricts innate immune responses to virus infection and DNA. Elife.

[7] Figueiredo VC, Markworth JF, Durainayagam BR, Pileggi CA, Roy NC, Barnett MPG, Cameron-Smith D (2016). Impaired ribosome biogenesis and skeletal muscle growth in a murine model of inflammatory bowel disease. Inflamm Bowel Dis.

[8] Galloway A, Kaskar A, Ditsova D, Atrih A, Yoshikawa H, Gomez-Moreira C, Suska O, Warminski M, Grzela R, Lamond AI (2021). Upregulation of RNA cap methyltransferase RNMT drives ribosome biogenesis during T cell activation. Nucleic Acids Res.

[9] Gabut M, Bourdelais F, Durand S (2020). Ribosome and translational control in stem cells. Cells-Basel.

[10] Lafontaine DL, Tollervey D (2001). The function and synthesis of ribosomes. Nat Rev Mol Cell Biol.

[11] Elhamamsy AR, Metge BJ, Alsheikh HA, Shevde LA, Samant RS (2022). Ribosome biogenesis: a central player in cancer metastasis and therapeutic resistance. Cancer Res.

[12] Pecoraro A, Pagano M, Russo G, Russo A (2021). Ribosome biogenesis and cancer: overview on ribosomal proteins. Int J Mol Sci.

[13] Neben CL, Lay FD, Mao X, Tuzon CT, Merrill AE (2017). Ribosome biogenesis is dynamically regulated during osteoblast differentiation. Gene.

[14] Barbieri I, Tzelepis K, Pandolfini L, Shi J, Millán-Zambrano G, Robson SC, Aspris D, Migliori V, Bannister AJ, Han N (2017). Promoter-bound METTL3 maintains myeloid leukaemia by m^6^A-dependent translation control. Nature.

[15] Lee Y, Choe J, Park OH, Kim YK (2020). Molecular mechanisms driving mRNA degradation by m^6^A modification. Trends Genet.

[16] Roundtree IA, Evans ME, Pan T, He C (2017). Dynamic RNA modifications in gene expression regulation. Cell.

[17] Liu Q, Li M, Jiang L, Jiang R, Fu B (2019). METTL3 promotes experimental osteoarthritis development by regulating inflammatory response and apoptosis in chondrocyte. Biochem Biophys Res Commun.

[18] Mi B, Xiong Y, Yan C, Chen L, Xue H, Panayi AC, Hu L, Hu Y, Zhou W, Cao F, Liu G (2020). Methyltransferase-like 3-mediated *N*^6^-methyladenosine modification of miR-7212-5p drives osteoblast differentiation and fracture healing. J Cell Mol Med.

[19] Wu Y, Xie L, Wang M, Xiong Q, Guo Y, Liang Y, Li J, Sheng R, Deng P, Wang Y (2018). Mettl3-mediated m^6^A RNA methylation regulates the fate of bone marrow mesenchymal stem cells and osteoporosis. Nat Commun.

[20] Tian C, Huang Y, Li Q, Feng Z, Xu Q (2019). Mettl3 regulates osteogenic differentiation and alternative splicing of Vegfa in bone marrow mesenchymal stem cells. Int J Mol Sci.

[21] Zhang Y, Gu X, Li D, Cai L, Xu Q (2020). METTL3 regulates osteoblast differentiation and inflammatory response via Smad signaling and MAPK signaling. Int J Mol Sci.

[22] Kong Y, Zhang Y, Cai Y, Li D, Yi B, Xu Q (2022). METTL3 mediates osteoblast apoptosis by regulating endoplasmic reticulum stress during LPS-induced inflammation. Cell Signal.

[23] Shore D, Albert B (2022). Ribosome biogenesis and the cellular energy economy. Curr Biol.

[24] Madan B, Harmston N, Nallan G, Montoya A, Faull P, Petretto E, Virshup DM (2018). Temporal dynamics of Wnt-dependent transcriptome reveal an oncogenic Wnt/MYC/ribosome axis. J Clin Investig.

[25] Pfister AS, Kuhl M (2018). Of Wnts and ribosomes. Prog Mol Biol Transl Sci.

[26] Chen L, Gao Y, Xu S, Yuan J, Wang M, Li T, Gong J (2023). *N*^6^-methyladenosine reader YTHDF family in biological processes: structures, roles, and mechanisms. Front Immunol.

[27] Ai R, Li D, Shi L, Zhang X, Ding Z, Zhu Y, He Y (2022). Periodontitis induced by orthodontic wire ligature drives oral microflora dysbiosis and aggravates alveolar bone loss in an improved murine model. Front Microbiol.

[28] Slots J (2017). Periodontitis: facts, fallacies and the future. Periodontology.

[29] Liu J, Chen M, Ma L, Dang X, Du G (2021). piRNA-36741 regulates BMP2-mediated osteoblast differentiation via METTL3 controlled m^6^A modification. Aging (Albany NY).

[30] Huang M, Xu S, Liu L, Zhang M, Guo J, Yuan Y, Xu J, Chen X, Zou J (2021). m^6^A Methylation regulates osteoblastic differentiation and bone remodeling. Front Cell Dev Biol.

[31] Bassler J, Hurt E (2019). Eukaryotic ribosome assembly. Annu Rev Biochem.

[32] Prakash V, Carson BB, Feenstra JM, Dass RA, Sekyrova P, Hoshino A, Petersen J, Guo Y, Parks MM, Kurylo CM (2019). Ribosome biogenesis during cell cycle arrest fuels EMT in development and disease. Nat Commun.

[33] Ni C, Buszczak M (2023). Ribosome biogenesis and function in development and disease. Development.

[34] Trainor PA, Merrill AE (2014). Ribosome biogenesis in skeletal development and the pathogenesis of skeletal disorders. Biochimica et Biophysica Acta (BBA) Mol Basis Dis.

[35] Ianniello Z, Sorci M, Ceci GL, Iaiza A, Marchioni M, Tito C, Capuano E, Masciarelli S, Ottone T, Attrotto C (2021). New insight into the catalytic-dependent and -independent roles of METTL3 in sustaining aberrant translation in chronic myeloid leukemia. Cell Death Dis.

[36] Pelletier J, Thomas G, Volarevic S (2018). Ribosome biogenesis in cancer: new players and therapeutic avenues. Nat Rev Cancer.

[37] Karner CM, Long F (2018). Glucose metabolism in bone. Bone.

[38] Smith CO, Eliseev RA (2021). Energy metabolism during osteogenic differentiation: the role of Akt. Stem Cells Dev.

[39] Liang YX, Hu W, Jin ZY, Diao HL, Liu L, Yang Y, Fu T, Yang ZM (2020). Nucleolar stress regulates stromal-epithelial transition via NPM1 during decidualization. Reproduction.

[40] Szaflarski W, Leśniczak-Staszak M, Sowiński M, Ojha S, Aulas A, Dave D, Malla S, Anderson P, Ivanov P, Lyons SM (2022). Early rRNA processing is a stress-dependent regulatory event whose inhibition maintains nucleolar integrity. Nucleic Acids Res.

[41] Destefanis F, Manara V, Bellosta P (2020). Myc as a regulator of ribosome biogenesis and cell competition: a link to cancer. Int J Mol Sci.

[42] Morcelle C, Menoyo S, Morón-Duran FD, Tauler A, Kozma SC, Thomas G, Gentilella A (2019). Oncogenic MYC induces the impaired ribosome biogenesis checkpoint and stabilizes p53 independent of increased ribosome content. Cancer Res.

[43] Piazzi M, Bavelloni A, Gallo A, Faenza I, Blalock WL (2019). Signal transduction in ribosome biogenesis: a recipe to avoid disaster. Int J Mol Sci.

[44] Karner CM, Long F (2017). Wnt signaling and cellular metabolism in osteoblasts. Cell Mol Life Sci.

[45] Liu J, Xiao Q, Xiao J, Niu C, Li Y, Zhang X, Zhou Z, Shu G, Yin G (2022). Wnt/beta-catenin signalling: function, biological mechanisms, and therapeutic opportunities. Signal Transduct Target Ther.

[46] Maeda K, Kobayashi Y, Koide M, Uehara S, Okamoto M, Ishihara A, Kayama T, Saito M, Marumo K (2019). The regulation of bone metabolism and disorders by Wnt signaling. Int J Mol Sci.

[47] Zhou Y, Zeng P, Li Y, Zhang Z, Cui Q (2016). SRAMP: prediction of mammalian *N*^6^-methyladenosine(m^6^A) sites based on sequence-derived features. Nucleic Acids Res.

[48] Zou Z, Sepich-Poore C, Zhou X, Wei J, He C (2023). The mechanism underlying redundant functions of the YTHDF proteins. Genome Biol.

[49] Xu Q, Tien Y, Shi Y, Chen S, Zhu Y, Huang X, Huang C, Zhao W, Yin X (2022). METTL3 promotes intrahepatic cholangiocarcinoma progression by regulating IFIT2 expression in an m^6^A-YTHDF2-dependent manner. Oncogene.

[50] Chen JX, Chen DM, Wang D, Xiao Y, Zhu S, Xu XL (2023). METTL3/YTHDF2 m^6^A axis promotes the malignant progression of bladder cancer by epigenetically suppressing RRAS. Oncol Rep.

[51] Li F, Kennedy S, Hajian T, Gibson E, Seitova A, Xu C, Arrowsmith CH, Vedadi M (2016). A radioactivity-based assay for screening human m^6^A-RNA methyltransferase, METTL3-METTL14 complex, and demethylase ALKBH5. SLAS Discov.

