# Supplementary material for: METTL3 promotes osteoblast ribosome biogenesis and alleviates periodontitis
Source: Clin Epigenetics. 2024 Jan 24;16:18. doi: 10.1186/s13148-024-01628-8 (PMC10809637; doi:10.1186/s13148-024-01628-8)
Supplement: Supplementary file 1 — Additional file 1: Figure S1. The effect of METTL3 on the rRNA stability. A The rRNA expression in METTL3-kncokdown cells after treating with 5 μg/mL ActD for 0–8 h was measured by qRT-PCR. n = 3. Figure S2. A, B The level of ROS was measured after METTL3 knockdown. n = 3. All data represent the mean ± SD. Figure S3. The effect of METTL3 knockdown on nucleolus, mTOR-Akt, and p53 pathway. A, B The nucleolar morphology of osteoblasts under LPS and osteogenic induction 3 days was assessed by immunocytochemistry. 20 nM actinomycin D (ActD) was the positive control of nucleolar stress. C The nucleolar number was detected by AgNOR staining. D, E The activation of p53 and AKT-mTOR signaling were examined by western blotting. *P < 0.05; **P < 0.01; ***P < 0.001. Figure S4. The effect of CHIR on the expression of Dkk3 and Sostdc1 in METTL3 knockdown cells. A, B The shCTRL and shMETTL3 cells were stimulated by LPS and osteogenic induction medium with or without CHIR. The mRNA expression of Dkk3 and Sostdc1 was detected by RT-qPCR. n = 3. All data represent the mean ± SD. *P < 0.05; ***P < 0.001. Figure S5. The effect of SAH and CHIR in periodontitis mice. A The proteins were evaluated in LPS-stimulated cells after stimulating with 5 μM SAH and 3 μM CHIR for 3 days. B Masson staining images of the periodontium. All data represent the mean ± SD. *P < 0.05; **P < 0.01. Table S1. Primer sequences for qRT-PCR. Table S2. Polysome profiling buffer. [file 13148_2024_1628_MOESM1_ESM.docx]

**Supplementary Information**

**METTL3 promotes osteoblast ribosome biogenesis and alleviates periodontitis**

Yiwen Zhang, Yiping Kong, Zhanqi Zhang, Jinlin He, Yongjie Cai, Yiqing Zhao, and Qiong Xu


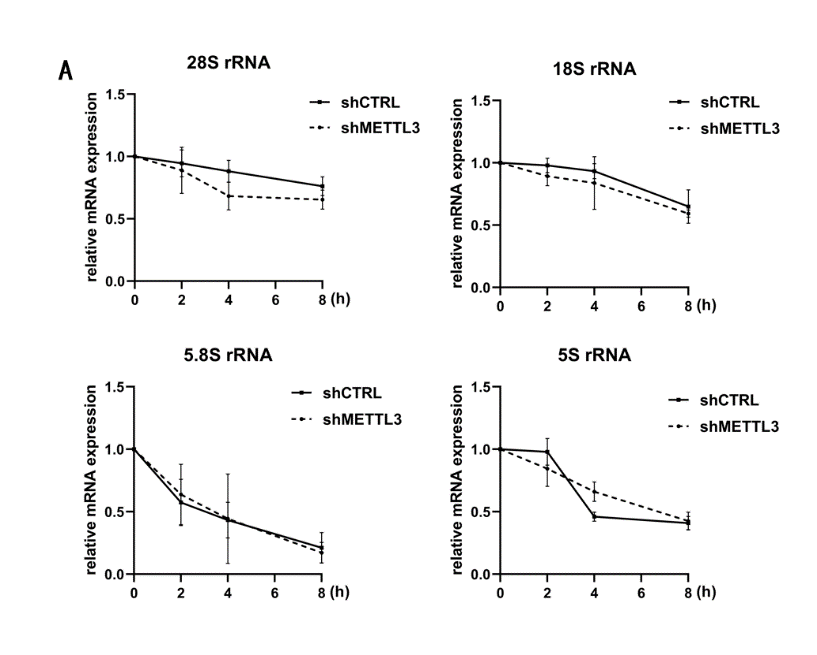


Figure S1. The effect of METTL3 on the rRNA stability. (A) The rRNA expression in METTL3-kncokdown cells after treating with 5 μg/mL ActD for 0-8 hours was measured by qRT-PCR. n=3.


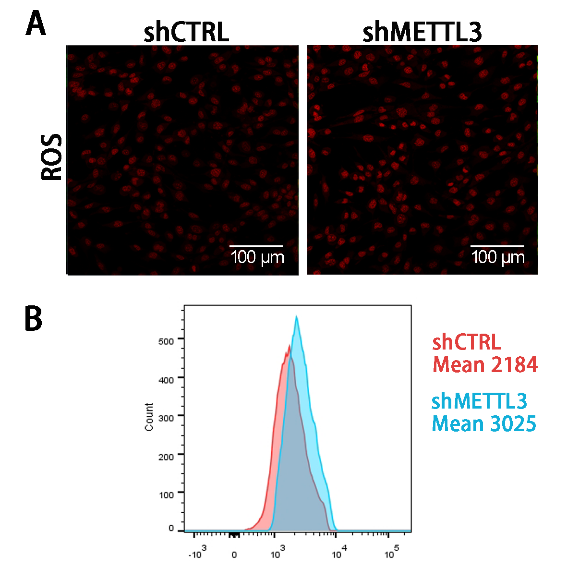


Figure S2. (A, B) The level of ROS was measured after METTL3 knockdown. n=3. All data represent the mean ± SD.


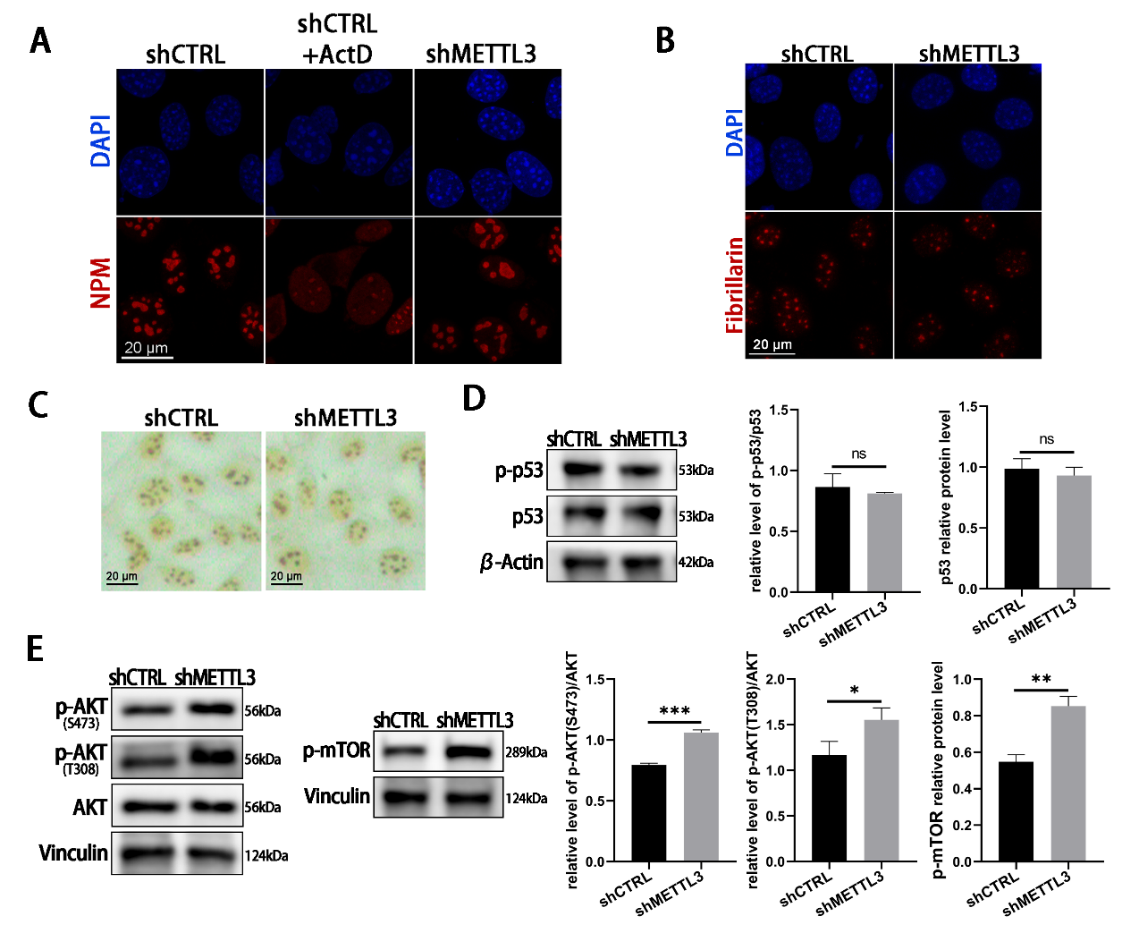


Figure S3. The effect of METTL3 knockdown on nucleolus, mTOR-Akt, and p53 pathway. (A, B) The nucleolar morphology of osteoblasts under LPS and osteogenic induction 3 days was assessed by immunocytochemistry. 20nM actinomycin D (ActD) was the positive control of nucleolar stress. (C) The nucleolar number was detected by AgNOR staining. (D, E) The activation of p53 and AKT-mTOR signaling were examined by western blotting. * *P* < 0.05, ** *P* < 0.01, *** *P* < 0.001.


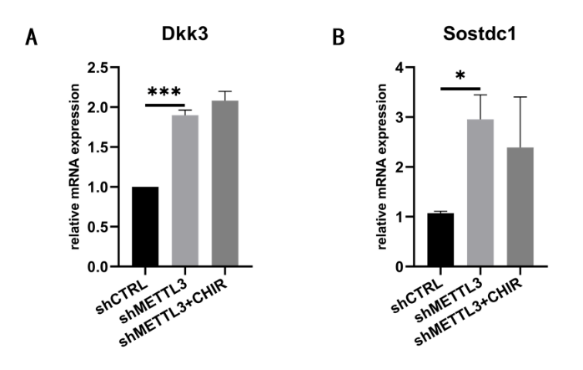


Figure S4. The effect of CHIR on the expression of Dkk3 and Sostdc1 in METTL3 knockdown cells. (A, B) The shCTRL and shMETTL3 cells were stimulated by LPS and osteogenic induction medium with or without CHIR. The mRNA expression of Dkk3 and Sostdc1 was detected by RT-qPCR. n=3. All data represent the mean ± SD. * *P* < 0.05, *** *P* < 0.001.


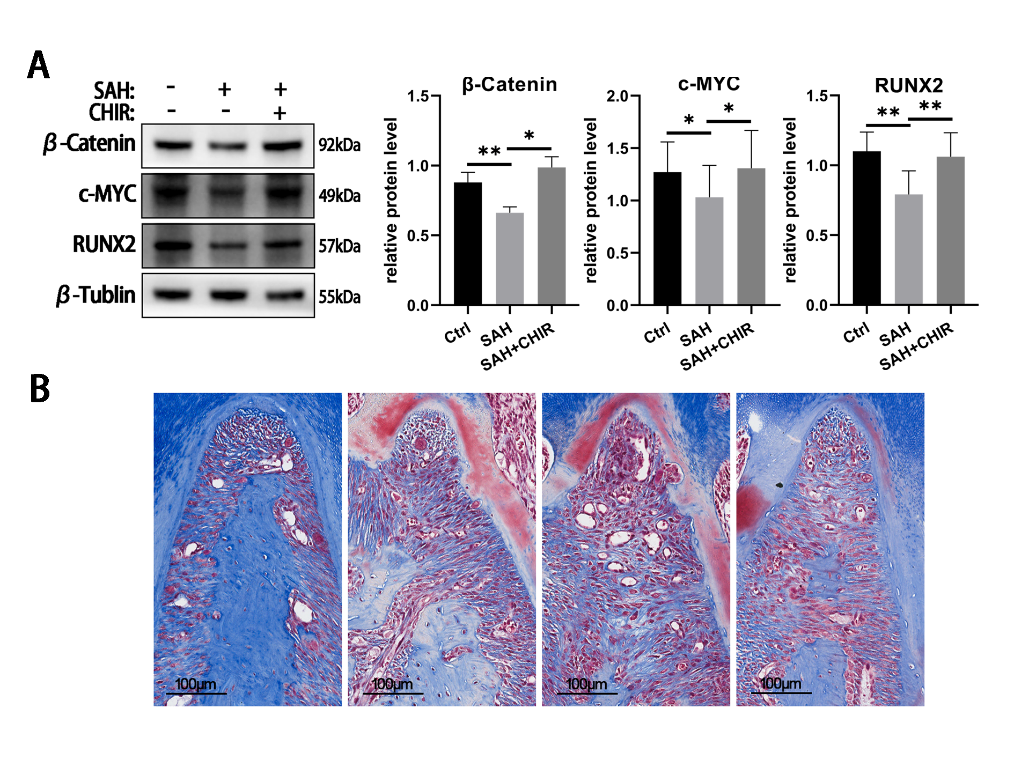


Figure S5. The effect of SAH and CHIR in periodontitis mice. (A) The proteins were evaluated in LPS-stimulated cells after stimulating with 5 μM SAH and 3 μM CHIR for 3 days. (B) Masson staining images of the periodontium. All data represent the mean ± SD. * *P* < 0.05, ** *P* < 0.01.

Table S1. Primer sequences for qRT-PCR

| Gene | Forward Primer | Reverse Primer | |
| --- | --- | --- | --- |
| c-Myc | ATGCCCCTCAACGTGAACTTC | | GTCGCAGATGAAATAGGGCTG |
| Dkk3 | CAGCTCTCAACTACCCTCAGG | | ACCTCAGAGGACGTTTTAGCA |
| Sostdc1 | GCGGTGTGTCAACGACAAGA | | GACCACGGTGATTTTGTAGGT |
| 45S rRNA | GCTTGTTTCTCCCGATTGC | | CGCGAACCACTGAGAAAAGT |
| 28S rRNA | TCATCAGACCCCAGAAAAGG | | GATTCGGCAGGTGAGTTGTT |
| 18S rRNA | CGGCTACCACATCCAAGGAA | | GCTGGAATTACCGCGGCT |
| 5.8S rRNA | ACTCTTAGCGGTGGATCACTC | | AAGCGACGCTCAGACAGG |
| 5S rRNA | GGCCATACCACCCTGAACGC | | CAGCACCCGGTATTCCCAGG |
| Atp5a1 | TCTCCATGCCTCTAACACTCG | | CCAGGTCAACAGACGTGTCAG |
| Uqcrc2 | AAAGTTGCCCCGAAGGTTAAA | | CAGAGAAGCAATCACCAAACCA |
| Sdha | GAACACTCCAAAAACAGACCTGC | | TCCACCACTGGGTATTGAGTAG |
| Ndufa7 | TCCGCTACTCGCGTTATCCA | | GATTGAGGGAGGCACAACTTC |
| Cox6c | ACAGATGCGTGGTCTTCTGG | | ACGCCTTCTTTCTTGGCTCAG |
| Rps12 | GCCCATGTATGTCAAGCTGGT | | CCCATTCCCCTAGTTTCTTGTTG |
| Rpl17 | ATGGTTCGCTACTCTCTTGACC | | AGTGAACACGAAGGTTTGACC |
| Rpl21 | GTGCTACCACGGCAAAACC | | TGTCTCTGCTCTTTGAGTGCT |
| β-actin | CATACCCAAGAAGGAAGGCTGG | | GCTATGTTGCTCTAGACTTCGAC |

Table S2. Polysome profiling buffer

| Buffer | Component |
| --- | --- |
| Lysis buffer | 5 mM Tris-HCl pH 7.5, 2.5 mM MgCl_2_, 1.5 mM NaCl, 1x protease inhibitor cocktail, 100 μg/mL cycloheximide, 2 mM DTT, 100 units RNase inhibitor, 1% Triton X-100 and 0.5% Sodium Deoxycholate |
| Sucrose buffer | 20 mM HEPES-KOH pH 7.6, 5 mM MgCl_2_, 100 mM NaCl, 10 units/mL RNase inhibitor, and 10 μg/mL cycloheximide |
